# Quantitative Proteomic Analysis of Human Embryonic Stem Cell Differentiation by 8-Plex iTRAQ Labelling

**DOI:** 10.1371/journal.pone.0038532

**Published:** 2012-06-18

**Authors:** Mahdieh Jadaliha, Hyoung-Joo Lee, Mohammad Pakzad, Ali Fathi, Seul-Ki Jeong, Sang-Yun Cho, Hossein Baharvand, Young-Ki Paik, Ghasem Hosseini Salekdeh

**Affiliations:** 1 Department of Molecular Systems Biology, Cell Science Research Center, Royan Institute for Stem Cell Biology and Technology, ACECR, Tehran, Iran; 2 Department of Biotechnology, College of Science, University of Tehran, Tehran, Iran; 3 Department of Biochemistry, Yonsei Proteome Research Center and Biomedical Proteome Research Center, Yonsei University, Sudaemoon-Ku, Seoul, Korea; 4 Department of Stem Cells and Developmental Biology, Cell Science Research Center, Royan Institute for Stem Cell Biology and Technology, ACECR, Tehran, Iran; 5 Department of Developmental Biology, University of Science and Culture, ACECR, Tehran, Iran; 6 Department of Systems Biology, Agricultural Biotechnology Research Institute of Iran, Karaj, Iran; University of Sao Paulo - USP, Brazil

## Abstract

Analysis of gene expression to define molecular mechanisms and pathways involved in human embryonic stem cells (hESCs) proliferation and differentiations has allowed for further deciphering of the self-renewal and pluripotency characteristics of hESC. Proteins associated with hESCs were discovered through isobaric tags for relative and absolute quantification (iTRAQ). Undifferentiated hESCs and hESCs in different stages of spontaneous differentiation by embryoid body (EB) formation were analyzed. Using the iTRAQ approach, we identified 156 differentially expressed proteins involved in cell proliferation, apoptosis, transcription, translation, mRNA processing, and protein synthesis. Proteins involved in nucleic acid binding, protein synthesis, and integrin signaling were downregulated during differentiation, whereas cytoskeleton proteins were upregulated. The present findings added insight to our understanding of the mechanisms involved in hESC proliferation and differentiation.

## Introduction

Human embryonic stem cells (hESCs) are pluripotent cells that have the potential to form any cell type and can be propagated in an undifferentiated state in vitro. Their exceptional properties mean they have tremendous potential for developmental biology, drug screening, functional genomics, and regenerative medicine. Developing reliable and reproducible protocols to differentiate hESCs into specific cell types and their transplantation into humans will require a detailed understanding of the molecular mechanisms that maintain the undifferentiated and pluripotent nature of hESCs.

Factors involved in hESCs self-renewal and pluripotency have been described [1], [2], [3], [4], [5], [6], and the overexpression of some of these factors in somatic cells has reprogrammed them into induced pluripotent stem cells (iPSCs), similar to hESCs. However, the genes and mechanisms that maintain the undifferentiated and pluripotent nature of hESCs are still largely unknown.

The proliferation and differentiation of embryonic stem cells (ESCs) are highly coordinated events that involve myriads of genes. A comprehensive profile of ESC gene expression can provide novel insights into the biology of these cells. Transcriptome analysis (i.e., microarray analysis) has shown tremendous potential for the analysis of ESC function and differentiation [7], [8], [9], but gene expression at the transcript level may not correlate well with its expression at the protein level due to alternative splicing, mRNA degradation, and posttranslational modifications such as phosphorylation and protein degradation. These concerns suggest that proteome analysis of ESCs can provide invaluable insights into pathways activated during ESC proliferation and differentiation.

Proteomic tools are valuable in studying ESC differentiation and elucidating the underlying molecular mechanisms [10], [11], [12]. Recent advances in state-of-the-art mass spectrometry (MS) techniques have demonstrated that MS-based quantitative proteomics approaches can significantly contribute to identifying proteins involved in ESC proliferation and differentiation.

Stable isotope labeling with amino acids in cell culture (SILAC) is also a powerful approach for quantitative proteomics [13] that has been used to comprehensively analyze self-renewing versus differentiating cells of two distinct hESC lines [14].

Another popular in vitro labeling method is the isobaric tag for relative and absolute quantitation (iTRAQ) reagent. A 4-plex iTRAQ has been used to analyze hESCs, mouse ES, and EC cells during differentiation by a 4-plex iTRAQ [15], [16]. Neural development from hESCs has been studied using an 8-plex iTRAQ reagent. A study of the progression of neural development from hESCs generated a catalog of approximately 1200 proteins and their relative quantitative expression patterns, which included several that changed expression levels during differentiation [17].

This study applied the 8-plex iTRAQ system to analyze hESCs during the differentiation of embryoid bodies (EB), as this system has the capability to compare several time points during a single experiment. The expression profiles for 1032 proteins during EB differentiation were analyzed and we identified 156 proteins that exhibited statistically significant changes in expression levels during differentiation.

## Materials and Methods

### Cell Culture

The hESC line, Royan H5, with normal karyotypes (46 XX) at passages 40–50 was used in this experiment. Briefly, the cells were first cultured on mouse embryonic fibroblasts (MEF), inactivated by mitomycin C (Sigma; M0503) [18]. The hESCs were then passaged and maintained under feeder-free conditions for 25–30 passages as described previously [19]. Then, ideal colonies were mechanically dissected into small pieces and replated on matrigel-coated dishes containing hESC medium (2 mM L-glutamine (Gibco; 25030-024), 0.1 mM β–mercaptoethanol (Sigma; M7522), DMEM/F12 medium (Gibco;21331-020) supplemented with 20% knock-out serum replacement (KSR, Gibco; 10828-028), 1% nonessential amino acid (Gibco; 11140-035), 100 units/ml penicillin and 100 µg/ml streptomycin (Gibco; 15070-063), 100 ng/mL basic-fibroblast growth factor (bFGF, Sigma; F0291)). The cells were grown in 5% CO2 at 95% humidity and the hESC medium was changed every day. The cells were further passaged as small clumps (100–500 cells) every 6–7 days after enzymatic treatment (2 mg/ml of dispase) and mechanical dissociation using a cell scraper by gently pipetting. To promote differentiation, hESCs were first cultured in suspension in ESC medium without KSR and containing fetal bovine serum (FBS) (ES-qualified; Gibco 16141-079), where they developed into multicellular aggregates called embryoid bodies (EBs). The EBs were cultured in suspension for 12 days and then plated onto gelatin-coated dishes for 8 days in the same medium to form a pool of spontaneously differentiated cells. Samples from undifferentiated hESCs and EBs at days 6 (EB6), 12 (EB12), and 20 (EB20) were collected for proteomics analysis.

To evaluate the percentage of undifferentiated hESCs, we analyzed the expression of key hESC markers including Nanog, Oct-4, SSEA-4, Tra-1-60, Tra-1-81, and SSEA-4 and Tra-1-60 or Tra-1-81 using two-color flow cytometry as previously described [11]. The analysis was performed by BD-FACS Caliber Flow Cytometer (Becton Dickinson) using following primary antibodies: anti-SSEA-4 (1∶50, ChemiconMAB4304) hOct-4 (1∶50, R&D MAB1759), Tra-1-60 (1∶20, Chemicon MAB4360), Tra-1-81 (1∶20, Chemicon MAB4381) and Nanog (1∶100, R&D MAB1994). Data from three independent replicate were analyzed by WinMDI software (version 2.8). Karyotype analysis and alkaline phosphatase staining was performed as described [11], [20].

### Protein Preparation and iTRAQ Isobaric Labeling

Samples of hESCs with at least 6×10^6^ cells in each of the three replicates from hESCs and differentiated derivatives at 6, 12, and 20 days after the initiation of differentiation were homogenized by sonication in 400 µl lysis buffer that consisted of 8 M urea, 4% w/v CHAPS, and one protease inhibitor tablet per 50 ml (Complete Protease Inhibitor Cocktail; Roche, Mannheim, Germany) on ice for 1 minute, with 2-second pulses every 2 seconds. The samples were then vortexed for 30 minutes at room temperature. Insoluble debris were pelleted by centrifugation at about 100,000×g (98,235×g) for 60 minutes at 4°C. The supernatant protein was quantified by the Bradford Assay Kit (BioRad, Hercules, CA) using bovine serum albumin as a standard. A total of 200 µg of each sample was reduced and denatured. The cysteines were then blocked as described in the 8-Plex iTRAQ protocol (Applied BioSystems, Foster City, CA). Each sample was digested with 20 µl of 0.25 µg/µl sequencing-grade modified trypsin solution (1∶20; Promega, Madison, WI) at 37°C, overnight. Samples were dried in a centrifugal vacuum concentrator, reconstituted with 30 µl dissolution buffer, and acidified with 0.1% formic acid to a pH of 2. The peptides were desalted with an Oasis HLB column (Waters, Milford, MA) and labeled with the iTRAQ tags (113–121 m/z): ESC (iTRAQ 113 and 117); EB6 (iTRAQ 114 and 118); EB12 (iTRAQ 115 and 119); and EB20 (iTRAQ 116 and 121). The labeled samples were then dried.

### Off-line Strong Cation Exchange (SCX) Chromatography and On-line Nano-LC ESI-MS/MS Analysis

**Figure 1:**
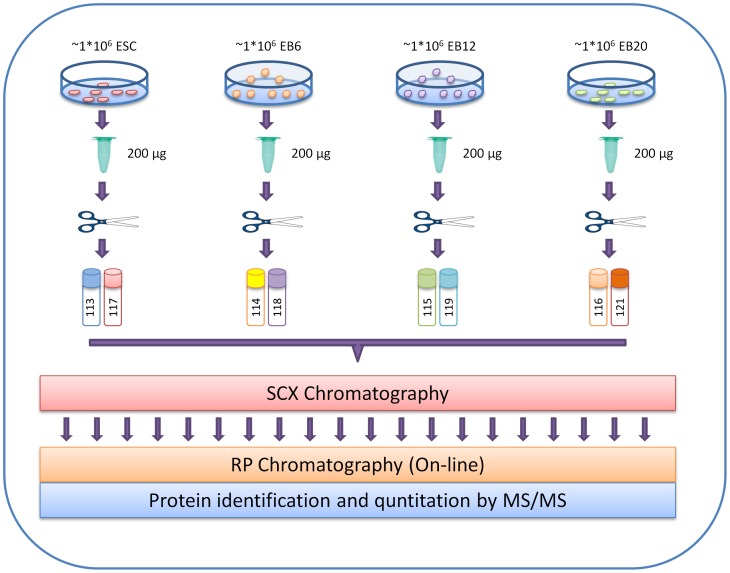
Experimental design of proteome analysis of hESCs using iTRAQ labeling. Samples from undifferentiated hESCs and EBs at days 6, 12, and 20 were collected in three biological replicates. Similar amounts of proteins were digested into peptides using trypsin. Peptides were subsequently desalted and labeled with 8-plex iTRAQ reagents 113–121. Labeled peptides were pooled, fractionated into 20 SCX fractions, and then analyzed by reverse-phase LC-MS/MS.

The peptides were separated, complexity was reduced, and all salts and urea were removed by pooling and injecting the acidified samples onto off-line strong cation exchange chromatography (SCX) columns. A total of 20 fractions were collected and dried by speed vacuum. Further separation was achieved by reverse-phase HPLC (Eksigent, Dublin, CA) that interfaced on-line to a QSTAR Elite mass spectrometer (Applied BioSystems). The mass spectrometer was operated in an information-dependent acquisition mode, whereby, following the interrogation of MS data (m/z 350–2000) using a 1-second survey scan, ions were selected for MS/MS analysis based on their intensity (>20 cpm) and charge state (+2, +3, and +4). Four product ion scans were set from each survey scan. Statistical evidence of differential expression of proteins was gained by performing two experiments for each biological replicate as described above (Figure 1).

### Data analysis and Interpretation

Relative abundances were quantitated, and peptides and proteins were identified with Protein-Pilot TM Software 2.0 (Applied BioSystems). Each MS/MS spectrum was searched for the *Homo sapiens* species in the UniProt database. The relative amount of a peptide in each sample was calculated by dividing the peak areas observed at 114.1, 115.1, and 116.1 m/z by those observed at 113.1 and 118.1 m/z, while those observed at 119.1 and 121.1 m/z were divided by that observed at 117.1 m/z. The peptides that did not have an iTRAQ modification were excluded. In addition, to avoid protein inference problem for proteins with high degree of sequence similarities, "Shared peptides", similar peptide sequence belongs to more than one protein reported in the results were excluded. The logarithm of each ratio was evaluated by a one-sample, unpaired t-test to determine statistical significance. Total significant proteins were clustered by the k-means clustering method with MATLAB version 7.3. The number of correct clusters was determined by measuring the average of intracluster and intercluster distances based on the similarity of a gene to the genes in its own cluster as compared to genes in other clusters [21], [22].

### Western Blot Analysis

Samples of 50 µg of proteins from three biological replicates were separated by 12% SDS-PAGE electrophoresis at 120 V for 1 hour with a Mini-PROTEAN 3 electrophoresis cell (Bio-Rad). The proteins were transferred to a PVDF membrane (Amersham, Uppsala, Sweden) by semi-dry blotting (Bio-Rad) with Dunn carbonate transfer buffer (10 mM NaCHO_3_, 3 mM Na_2_CO_3_, 20% methanol). The membranes were blocked for 1.5 hours using a western blocker solution (Sigma, St. Louis, MO, W0138). Each membrane was incubated overnight at 4°C with one of these primary monoclonal antibodies: anti-ERP29 (1∶4000; Abcam); anti-NPM1 (1∶1000; Sigma); anti-HSC70 (1∶10000; Stressgen); anti-CALU (1∶4000; Santa Cruz Biotechnology, Santa Cruz, CA); or anti-STMN1 (1∶2000; Abcam). Next, the membranes were incubated for 2 hours at room temperature with the following peroxidase-conjugated secondary antibodies: anti-mouse (1∶180,000; Sigma, A9044); anti-rat (1∶160,000; Sigma, A5795), and anti-rabbit (1∶160,000; Sigma, A2074). Finally, the blots were visualized with ECL detection reagent (Sigma, CPS-1-120). The films were then scanned with a GS-800 densitometer (Bio-Rad), and quantitative analysis was performed with UVI bandmap software (UVItec, Cambridge, UK). The uniformity of the amounts of proteins loaded on the gels was investigated by staining the membranes with Fast Green (Sigma, F7252).

## Results and Discussion

### Cell Characterization

The hESCs grew as compact colonies with a high nuclear to cytoplasmic ratio and prominent nucleoli (Figure S1A and S1B). The colony showed a typical undifferentiated morphology with a distinct boundary, and each cell presents a compact morphology with a high nucleus to cytoplasmic ratio, that contains prominent nucleoli typical of undifferentiated hESCs. Moreover, the hESC line had a normal karyotype (46 XX) (Figure S1 C). To induce differentiation, hESCs were cultured as EBs (Figure S1 D-G). The high percentage of undifferentiated hESCs was confirmed by expression analysis of key hESC markers including Nanog, Oct-4, SSEA-4, Tra-1-60, Tra-1-81 (Figure S1 H and Table S1).

### Proteome Analysis

We used an 8-plex iTRAQ system to analyze the proteomes of hESCs during proliferation and at different stages of differentiation in three biological replicates. For each biological replicate, the experiments were repeated twice. Protein lysates from ESCs and EBs on days 6, 12, and 20 after initiation of differentiation were used for iTRAQ labeling. These proteins represented the different stages of spontaneous differentiation. The proteins were labeled with eight different iTRAQ reagents (113–121) as follows: iTRAQ 113 and 117 for ESC; iTRAQ114 and118 for EB6; iTRAQ115 and 119 for EB12; and iTRAQ 116 and 121 for EB20. Labeled proteins were then pooled and fractionized by SCX. A total of 20 SCX fractions were analyzed by reverse-phase LC-MS/MS as described in Materials and Methods. MS/MS spectra were searched and quantitated.

A total of 2153 unique proteins were identified, of which 1648 proteins with quantitation ratios were considered for further analyses. By combining six replicates, 1032 nonredundant proteins were confidently identified and quantified by the criterion of unused protein score <1.3 (95% CI) per experiment. The complete list of all proteins identified from the three biologically independent replicates and the confidence scores is provided in Table S2.

Proteins with statistically significant changes during differentiation were identified by filtering according to these criteria: 1) they had to be present in at three replicates, including two biological replicates; 2) changes between stages had to be statistically significant (P<0.05); and 3) fold change had to be greater than 1.2. This approach allowed us to select 159 differentially expressed proteins for further analysis.

**Figure 2:**
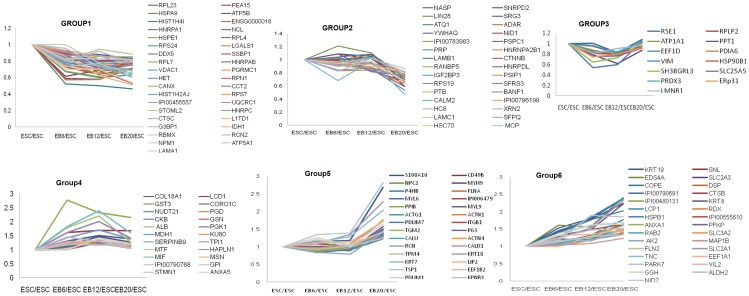
K-mean clusters of differentially expressed proteins. These proteins could be clustered into six different groups with clusters 1–3 representing downregulated proteins and clusters 4–6 representing upregulated proteins. In cluster 1, the abundance of 37 proteins decreased significantly at all three stages. In cluster 2, 30 proteins were downregulated at EB20, the late stage of differentiation. In cluster 3, the expression levels of 13 protein decreased at EB6 and EB12. In cluster 4, 22 proteins showed higher abundance in EB6 and EB12. In cluster 5, 28 proteins significantly increased in abundance at EB20 and in cluster 6, 29 proteins had higher abundance in all three stages that was more pronounced at EB20.

Out of 156 differentially expressed proteins, 81 were found to be downregulated and 79 upregulated in at least one EB compared to ESCs (Table S2 and S3). These proteins could be clustered into six different groups (Figure 2, Table S4), which represented downregulated proteins in clusters 1–3 and upregulated proteins in clusters 4–6. In cluster 1, the abundance of 37 proteins decreased significantly at all three stages. In cluster 2, 30 proteins were downregulated at EB20 (late stage of differentiation) and included such proteins as Lin28, the widely used marker of pluripotency. In cluster 3, the expression levels of 13 protein decreased at EB6 and EB12, however these did not change significantly at EB20. In cluster 4, 22 proteins were upregulated mainly in EB6 and EB12, with more pronounced upregulation in EB12. In cluster 5, 28 proteins significantly increased in abundance at EB20 but did not change at EB6 and EB12. In cluster 6, 29 proteins had upregulated expression in all three stages, but was more pronounced at EB20.

**Figure 3:**
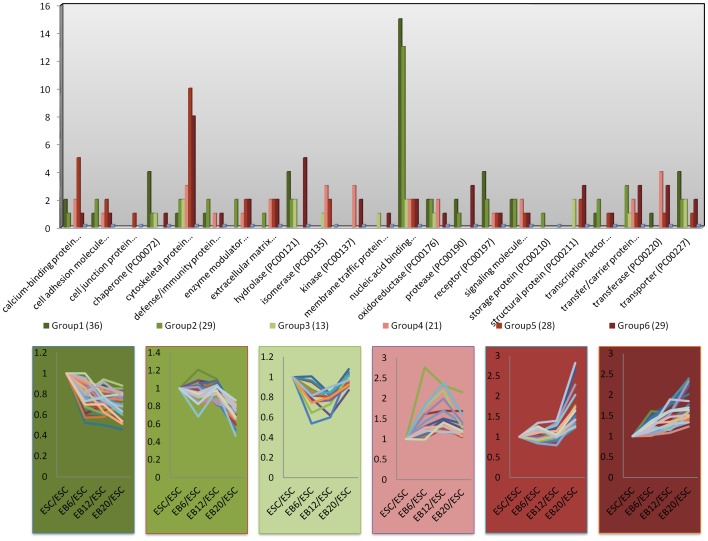
The functional analysis of 6 k-mean clusters. This list of proteins was employed to identify significantly activated pathways by comparing their functional annotations according to the PANTHER classification systems. Proteins involved in nucleic acid binding, protein synthesis, and signaling (particularly, proteins involved in integrin signaling) were enriched in the downregulated protein clusters (clusters 1, 2, and 3), whereas the upregulated proteins in clusters 4, 5, and 6 were enriched in proteins involved in cytoskeleton structure.

Functional analysis of expression clusters with Panther software (www.pantherdb.org) revealed that proteins involved in nucleic acid binding, protein synthesis, and signaling, (particularly integrin signaling), were enriched in the downregulated protein clusters (clusters 1, 2, and 3), whereas the upregulated proteins in clusters 4, 5, and 6 were enriched in proteins involved in cytoskeleton structure (Figure 3). These proteins are listed in Table S5.

### iTRAQ Results Confirmed by Western Blot Analysis

**Figure 4:**
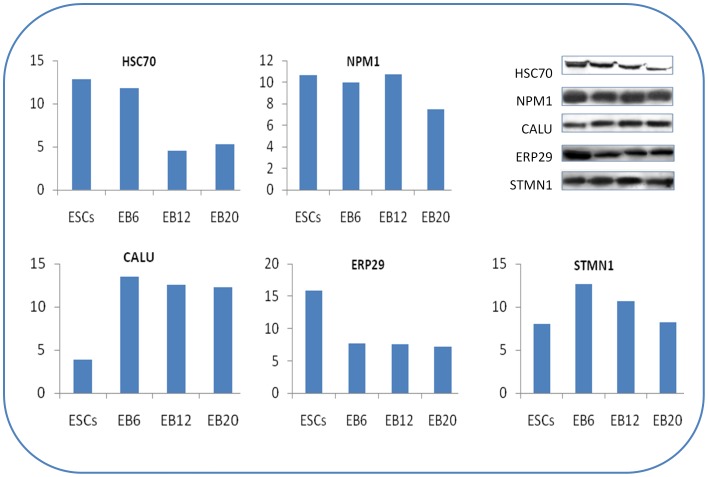
Western blot analysis of the total protein derived from Royan H5. Fifty micrograms of protein from three biological replicates extracted from three independent replications of hESCs and Dif-ESCs of Royan H5 were subjected to SDS-PAGE followed by Western blotting. ESCs and Dif-ESCs were analyzed with antibodies against CALU, ERP31, NPM1, HSC70, and STMN1. The y-axis represents the area in Western blot for different stages (x-axis). Protein bands were quantified using UVI bandmap software. Error bars represent the standard deviation of three measurements.

The iTRAQ results were confirmed by Western blot analysis, which examined the expression levels of CALU, ERP31, NPM1, HSC70, and STMN1 (Figure 4). The levels of HSC70, ERP29, and NPM1 decreased during differentiation while those of CALU and STMN1 increased. Western blot analysis confirmed the results of the iTRAQ analysis, although the fold changes obtained by the two approaches differed slightly from each other (Figure 4).

### ESCs Express Relatively High Levels of Nucleic Acid Binding Proteins

Proteins downregulated during differentiation included the nucleic acid binding proteins DEAD box RNA helicase (DDX5), scaffold attachment factor B1 (SAFB1), HIST1H2AJ, HNRNPA2B1, HNRPA1, HNRPAB, nucleolin (NCL), RBMX, SSBP1, adenosine deaminase (ADAR1), HNRPDL, insulin-like growth factor 2 mRNA binding protein 3 (IGF2BP3), LIN28, PSPC1, SNRPD2, XRN2, and the widely used marker of pluripotency, LIN28. The downregulated protein IGF2BP3 is expressed mainly in embryonic development and in some tumors, and has been demonstrated to promote cell proliferation by inducing translation of IGF-II mRNA in K562 leukemia cells [15]. The downregulated protein NCL is a major nucleolar protein that plays a role in many pathways and functions [23]. Downregulation of NCL has been shown to be detrimental to the growth of ESCs and to increase the rate of apoptosis, which suggests its importance in maintaining the self-renewal of ESCs [24]. Downregulated ADAR1 is responsible for RNA editing by site-specific deamination of adenosines [25]. Knockdown of ADAR1 globally affects gene expression in hESCs and results in significantly increased RNA expression levels of genes involved in differentiation and developmental processes, including neurogenesis [26]. ADAR1 has been suggested to play a role in regulating hESC early differentiation [26]. Members of the heterogeneous nuclear ribonucleoprotein (HNRNP) family, which included HNRNPA2B1, HNRPA1, HNRPAB, HNRPDL, HNRNPI, and RBMX were downregulated, which was consistent with several studies [10], [11], [12].

Several identified proteins are multifunctional and contain numerous highly conserved functional domains. The SAFB1 protein can bind both DNA and RNA and is involved in RNA processing and stress response. It contains a transcriptional repression domain, can bind certain hormone receptors to repress their activity [27], and may also be involved in development, growth regulation, and reproduction [28]. DDX5 protein regulates aspects of RNA expression, including replication, transcription and splicing, and is required for cell growth [29]. It has been demonstrated to be a novel co-activator for Runx2, and can inhibit the osteogenic differentiation of mesenchymal progenitor cells [30].

### EBs Increase Expression of Cytoskeleton-associated Proteins

The proteomes of EBs and ESCs exhibited differences in the expressions of cytoskeleton-pathway proteins. A total of 20 out of 26 cytoskeleton-associated proteins showed higher expression levels in EBs than ESCs. The altered proteins included CORO1C, GSN, MSN, ACTG1, CALD1, KRT18, KRT7, MYH9, MYL6, MYL9, PDLIM7, PDLIM1, TPM4, DSP, KRT19, KRT8, MAP1B, RDX, SNL, and VIL2. Cytoskeleton-associated proteins had downregulated expression in mouse, monkey, and human EB-mediated differentiation of ESCs [11], [12], [31]. The cell shape has been suggested to be a cue in the commitment process [32], and changes in cell shape may be transduced into a regulatory signal by several structures in the cell, including the actin cytoskeleton itself [33]. Mechanical tension can control the differentiation status of adult stromal stem cells through the actin filament complex [32].

### Regulation of Calcium-binding Proteins

Calcium-binding proteins that included CANX, RCN2, MCP, ANXA5, GSN, CALU, MYL6, MYL9, RCN, S100A10, and ANXA1 were significantly regulated. Calcium-binding proteins, such as calreticulin, pyruvate dehydrogenase complex, and the translationally-controlled tumor protein are regulated during neural differentiation of mouse ESCs [34], implying that Ca^2+^ may play an important role in hESC differentiation. The Ca^2+^ ion is a highly versatile intracellular signal that regulates many different cellular functions, including fertilization, cell cycle, apoptosis, muscle contraction, vision, and memory.

Intracellular Ca^2+^ homeostasis is maintained through a complex interplay between intracellular stores such as the endoplasmic reticulum and extracellular Ca^2+^ that enters the cell through various transporters on the plasma membrane. Calcium regulates gene expression by modulating transcription factors [35] and mediates posttranslational modifications by modulating protein kinases, phosphatases, and Ca^2+^-sensitive adenylate cyclases [36]. Calcium modulates intercellular communication through gap junctions and triggers the terminal differentiation programs of cells. However, the details of Ca^2+^ homeostasis and signaling, and the mechanism by which Ca^2+^ regulates Ca^2+^-related proteins during ESC differentiation remain to be determined.

### Ribosomal Proteins Downregulated

Ribosomal proteins, including RPL23, RPS24, RPL7, RPS7, RPLP2, and RPS19, were more abundant in undifferentiated ESCs than in differentiated cells. This finding has been supported by the observation that ESCs possess a surplus of free ribosomes as ribosomal subunits and single ribosomes that are recruited to actively translating polysomes during differentiation [37]. The protein synthesis capacity of ESCs has been suggested to be poised to allow rapid elevation of translation rate in response to differentiation signals [37].

### Proteins Involved in Integrin Signaling were more Abundant in Undifferentiated ESCs

Extracellular matrix (ECM) signaling is predominantly transmitted via cell membrane receptors of the integrin family [38]. The spatially and temporally controlled engagement of different integrin receptors during embryogenesis demonstrates their roles during commitment and lineage determination of early embryogenesis [39].

Several proteins involved in integrin signaling including CD49B, FLNA, ACTN4, ITGB1, FLN2, and EDS4A were more abundant in ESCs than in EBs. An in vitro loss-of-function approach based on b1 integrin-deficient ESCs found that integrin-dependent mechanisms were involved in the regulation of Wnt-1 and BMP-4 expression [40]. Integrin signaling engineered in mouse ESCs demonstrated the critical role of simultaneous signaling from identified integrins in maintaining pluripotency [41].

**Table 1:** Correlation between the expression patterns of differentially expressed proteins identified in this study and their corresponding mRNA at three different stages.

	*RNA*				
*Protein*	*Up*	*No change*	*Down*	*Uniformity of Pr-RNA pattern (%)*	*Uniformity of RNA-Pr pattern (%)*
**EB6/ESC**					
Up	**12**	20	6	31.58	33.33
No change	20	**60**	35	52.17	59.41
Down	4	21	**6**	19.35	12.77
*Total*				42.39	42.39
**EB12/ESC**					
Up	**24**	22	6	46.15	52.17
No change	15	**36**	34	42.35	42.86
Down	7	26	**14**	29.79	25.93
*Total*				40.22	40.22
**EB20/ESC**					
Up	**51**	24	6	62.96	68.92
No change	10	**14**	9	42.42	22.22
Down	13	25	**32**	45.71	68.09
*Total*				72.86	72.86

### The Correlation between mRNA and Protein Levels

Transcriptomics techniques (i.e., microarray) are powerful approaches to profile mRNA expression. The extent that changing mRNA expression patterns reflect corresponding changes in their cognate proteins is an important issue. We compared the expression patterns of proteins with the level of their corresponding mRNA previously analyzed by Fathi et al [11] were analyzed to determine the correspondence between protein and mRNA levels (Table 1 and Figure S2). Pearson’s method measured the correlations between 156 differentially expressed proteins and their corresponding mRNAs. The correlation between RNA and protein abundance levels was low (Figure S2). A trend of correlation between protein and mRNA changes was observed, but with many exceptions (Table 1).

### Conclusion

We applied an iTRAQ-based quantitative proteomics approach to characterize changes in the proteome pattern of hESCs during differentiation. Our findings add insight to the understanding of the mechanisms involved in hESC proliferation and differentiation. Large-scale iTRAQ labeling methods are limited because combining labeled samples from different stages may result in peptides that have been derived from different stages dominating over those specifically found in only one or two stages. This limits their identification in regular data-dependent acquisition of highly abundant ions, such as several major transcription factors (Oct-4, Nanog, and SOX2) not identified by this study. Possibly, these proteins are highly expressed only in ESCs, and the peptides from these proteins become diluted upon mixing labeled peptides from several stages of differentiation. This work provides experimental evidence of the contributions of several candidate proteins and mechanisms in the differentiation of hESCs, but the function of these proteins in self-renewal and differentiation still needs to be precisely clarified.

## Supporting Information

Figure S1 Characterization of undifferentiated hESCs (Royan H5) and differentiated EBs at different time points. (A) Phase contrast photographs of a Royan H5 colony grown under feeder-free conditions and its high magnification photo. (B) Expression of alkaline phosphatase. (C) The karyotype of Royan H5. (D–G) EB formation by generating some clamps from undifferentiated hESCs at day 0 (D), day 6 (E), day 12 (F), and day 20. (G) After 12 days in suspension, EBs were plated on 0.1% gelatin-coated plates in the same medium to form a pool of spontaneously differentiated cells. The percentages of undifferentiated and differentiated hESCs are shown in (H) by the use of BD-FACS Caliber flow cytometry (Becton Dickinson). Data from three independent replicates were analyzed by WinMDI software (version 2.8). Bar = 500 µm.
(DOCX)

Figure S2 The heat map and Pearson correlation of relative mRNA and protein abundance for stages EB6/ESC, EB12/ESC, and EB20/ESC. The correlation was calculated for 184 mRNA, which paired 156 significantly changed proteins (there were more than one mRNA data for some proteins). The heat map is divided into 6 blocks, considering 6 different k-mean groups.
(DOCX)

Table S1 Flow cytometric analysis of three replicates regarding five hESC markers, Oct4, Nanog, SSEA-4, Tra 1-60 and Tra 1-81. Three replicates are showing similar patterns and stem cell related proteins were being down-regulated during EB formation.
(DOCX)

Table S2 The list of identified differentially expressed proteins in different stages of EB6, EB12 and EB20 compared to ESC.
(DOCX)

Table S3 Quantification details of different repeats (for all and regulated proteins). The quantification data for different stages and different replicates (shown in yellow highlighted columns) has been used for t-test analysis and resulted in identification of regulated proteins considering three criteria mentioned before. This table includes: **N** which presents the rank of a protein relative to all other proteins in the list of detected proteins in each repeat; **Total** ProtScore is an indicator of the total amount of evidence for a detected protein. The Total ProtScore is calculated using all of the peptides detected for the proteins, and does not show the confidence percentage of the identification of a protein. **Unused** ProtScore is an indicator of the protein confidence for a detected protein which is calculated from the peptide confidence for peptides from spectra that have not already been used by other proteins. **%Cov** represents the percentage of matching amino acids to at least one identified peptide having confidence greater than 0 divided by the total number of amino acids in the protein sequence. **%Cov(50)** and **%Cov(95)** are the percentage of matching amino acids to at least one identified peptide having confidence greater than or equal to 50 and 95, respectively, divided by the total number of amino acids in the protein sequence. **Ratio** is the average ratio for the protein in EBs compared to ESCs, which is corrected for experimental bias. The iTRAQ tags are iTRAQ 113 and 117 for ESC, iTRAQ 114 and 118 for EB6, iTRAQ 115 and 119 for EB12 and iTRAQ 116 and 121 EB20. **P-value** is a measure of the certainty that the average ratio differs from one (this p-value is for each repeat and is different from t-test analysis for all 6 replicates which has been done to detect regulated proteins with shown data in *regulated proteins* sheet). The error factor (**EF)** is a measure of the error in the average ratio, representing the 95% confidence interval of the average iTRAQ ratio as (ratio×EF) – (ratio/EF).
(XLS)

Table S4 List of the proteins depicted in the figure 2, with their expression ratio and p-values.
(DOCX)

Table S5 Classification of differentially expressed proteins according their protein classes. Table contains the class of each protein and also protein classes enriched in the every k-means clusters.
(XLS)
