# Supplementary material for: Quantitative Proteomic Analysis of Human Embryonic Stem Cell Differentiation by 8-Plex iTRAQ Labelling
Source: PLoS One. 2012 Jun 18;7(6):e38532. doi: 10.1371/journal.pone.0038532 (PMC3377673; doi:10.1371/journal.pone.0038532)

**Figure S1**

Characterization of undifferentiated hESCs (Royan H5) and differentiated EBs at different time points. (A) Phase contrast photographs of a Royan H5 colony grown under feeder-free conditions and its high magnification photo. (B) Expression of alkaline phosphatase. (C) The karyotype of Royan H5. (D-G) EB formation by generating some clamps from undifferentiated hESCs at day 0 (D), day 6 (E), day 12 (F), and day 20. (G) After 12 days in suspension, EBs were plated on 0.1% gelatin-coated plates in the same medium to form a pool of spontaneously differentiated cells. The percentages of undifferentiated and differentiated hESCs are shown in (H) by the use of BD-FACS Caliber flow cytometry (Becton Dickinson). Data from three independent replicates were analyzed by WinMDI software (version 2.8). Bar = 500µm.


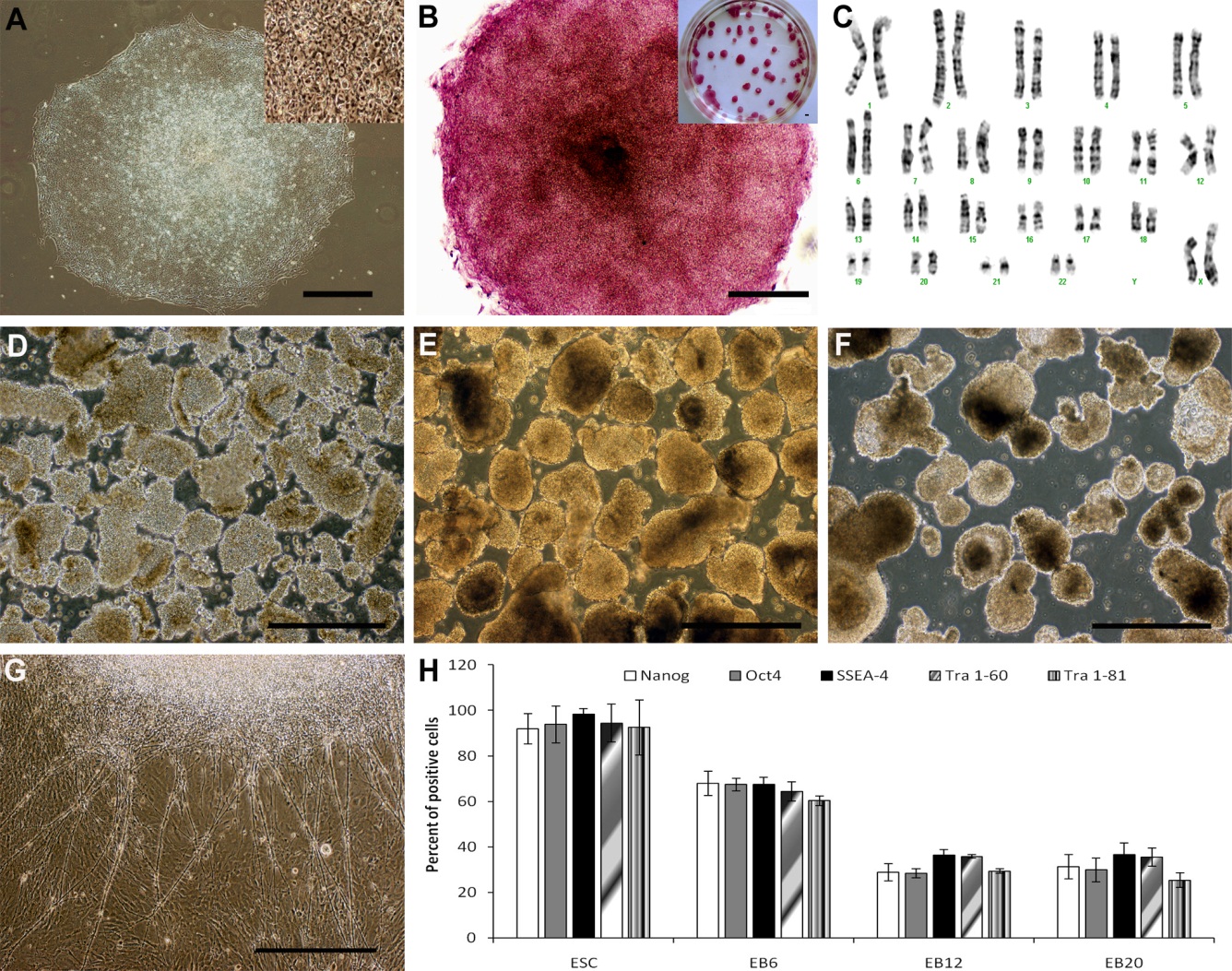

Supplement: Figure S1 — Characterization of undifferentiated hESCs (Royan H5) and differentiated EBs at different time points. (A) Phase contrast photographs of a Royan H5 colony grown under feeder-free conditions and its high magnification photo. (B) Expression of alkaline phosphatase. (C) The karyotype of Royan H5. (D–G) EB formation by generating some clamps from undifferentiated hESCs at day 0 (D), day 6 (E), day 12 (F), and day 20. (G) After 12 days in suspension, EBs were plated on 0.1% gelatin-coated plates in the same medium to form a pool of spontaneously differentiated cells. The percentages of undifferentiated and differentiated hESCs are shown in (H) by the use of BD-FACS Caliber flow cytometry (Becton Dickinson). Data from three independent replicates were analyzed by WinMDI software (version 2.8). Bar = 500 µm. (DOCX) [file pone.0038532.s001.docx]
