# Supplementary material for: Quantitative Proteomic Analysis of Human Embryonic Stem Cell Differentiation by 8-Plex iTRAQ Labelling
Source: PLoS One. 2012 Jun 18;7(6):e38532. doi: 10.1371/journal.pone.0038532 (PMC3377673; doi:10.1371/journal.pone.0038532)

**Figure S2**

The heat map and Pearson correlation of relative mRNA and protein abundance for stages EB6/ESC, EB12/ESC, and EB20/ESC. The correlation was calculated for 184 mRNA which paired 156 significantly changed proteins (there were more than one mRNA data for some proteins). The heat map is divided into 6 blocks considering 6 different k-mean groups.


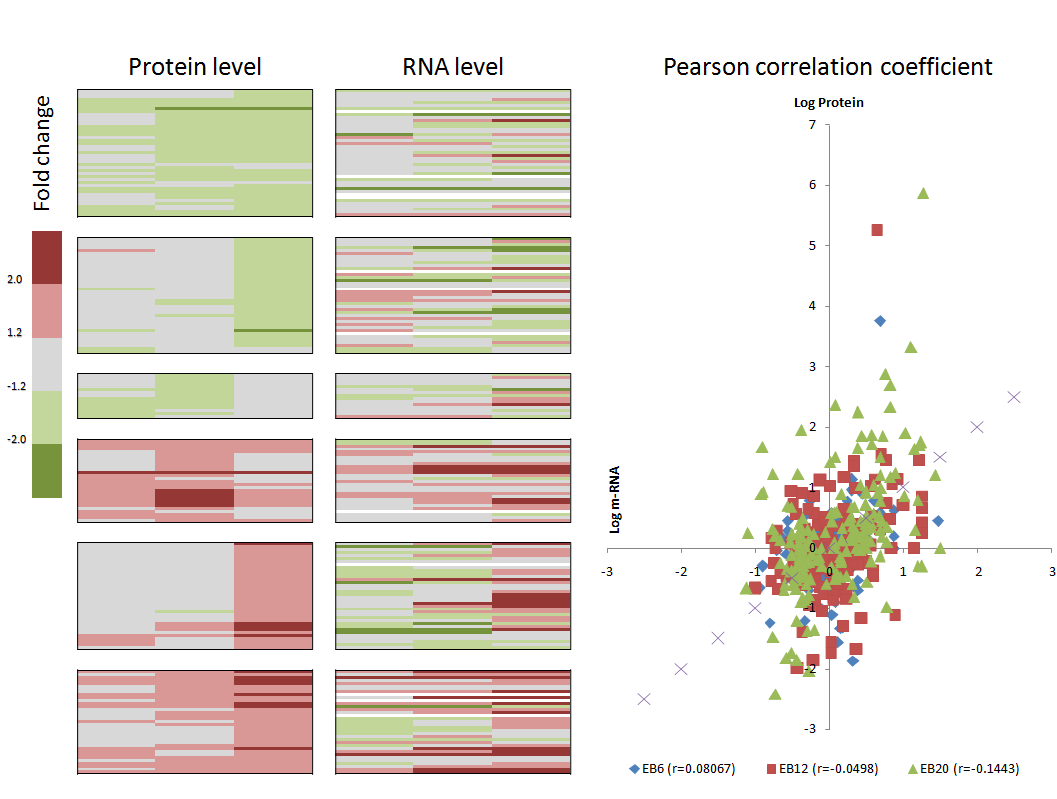

Supplement: Figure S2 — The heat map and Pearson correlation of relative mRNA and protein abundance for stages EB6/ESC, EB12/ESC, and EB20/ESC. The correlation was calculated for 184 mRNA, which paired 156 significantly changed proteins (there were more than one mRNA data for some proteins). The heat map is divided into 6 blocks, considering 6 different k-mean groups. (DOCX) [file pone.0038532.s002.docx]
