# Supplementary material for: Quantitative Proteomic Analysis of Human Embryonic Stem Cell Differentiation by 8-Plex iTRAQ Labelling
Source: PLoS One. 2012 Jun 18;7(6):e38532. doi: 10.1371/journal.pone.0038532 (PMC3377673; doi:10.1371/journal.pone.0038532)
Supplement: Table S1 — Flow cytometric analysis of three replicates regarding five hESC markers, Oct4, Nanog, SSEA-4, Tra 1-60 and Tra 1-81. Three replicates are showing similar patterns and stem cell related proteins were being down-regulated during EB formation. (DOCX) [file pone.0038532.s003.docx]

**Supplementary table 1**

Flow cytometric analysis of three replicates regarding five hESC markers, Oct4, Nanog, SSEA-4, Tra 1-60 and Tra 1-81. Three replicates are showing similar patterns and stem cell markers were being down-regulated during EB formation.

| Stage | Marker | R1 | R2 | R3 | Averege. | STDEV |
| --- | --- | --- | --- | --- | --- | --- |
| Day0 | SSEA-4 | 99.65 | 95.73 | 99.79 | 98.39 | 2.3047 |
|  | Tra 1-60 | 99.29 | 84.82 | 99.2 | 94.437 | 8.3284 |
|  | Tra 1-81 | 99.89 | 78.56 | 99.12 | 92.523 | 12.099 |
|  | Nanog | 99.48 | 89.67 | 86.63 | 91.927 | 6.7157 |
|  | Oct4 | 99.54 | 84.56 | 97.33 | 93.81 | 8.0866 |
|  |  |  |  |  |  |  |
| Day6 | SSEA-4 | 70.21 | 64.03 | 68.26 | 67.5 | 3.1593 |
|  | Tra 1-60 | 69.35 | 62.05 | 61.85 | 64.417 | 4.2736 |
|  | Tra 1-81 | 58.69 | 62.73 | 59.64 | 60.353 | 2.1124 |
|  | Nanog | 74.13 | 64.61 | 65.25 | 67.997 | 5.3213 |
|  | Oct4 | 70.45 | 65.55 | 66.16 | 67.387 | 2.6704 |
|  |  |  |  |  |  |  |
| Day12 | SSEA-4 | 34.11 | 36.05 | 39.15 | 36.437 | 2.5422 |
|  | Tra 1-60 | 36.02 | 34.95 | 36.43 | 35.8 | 0.7641 |
|  | Tra 1-81 | 29.16 | 28.49 | 30.57 | 29.407 | 1.0617 |
|  | Nanog | 31.15 | 24.63 | 31.15 | 28.977 | 3.7643 |
|  | Oct4 | 29.68 | 26.08 | 29.68 | 28.48 | 2.0785 |
|  |  |  |  |  |  |  |
| Day12+8 | SSEA-4 | 32.05 | 42.36 | 35.36 | 36.59 | 5.2639 |
|  | Tra 1-60 | 30.98 | 38.69 | 36.78 | 35.483 | 4.0152 |
|  | Tra 1-81 | 22.09 | 28.39 | 25.82 | 25.433 | 3.1677 |
|  | Nanog | 32.54 | 36.07 | 25.61 | 31.407 | 5.3213 |
|  | Oct4 | 36 | 26.72 | 27.02 | 29.913 | 5.2733 |
|  |  |  |  |  |  |  |
