# Supplementary material for: Quantitative Proteomic Analysis of Human Embryonic Stem Cell Differentiation by 8-Plex iTRAQ Labelling
Source: PLoS One. 2012 Jun 18;7(6):e38532. doi: 10.1371/journal.pone.0038532 (PMC3377673; doi:10.1371/journal.pone.0038532)
Supplement: Table S2 — The list of identified differentially expressed proteins in different stages of EB6, EB12 and EB20 compared to ESC. (DOCX) [file pone.0038532.s004.docx]

S**upplementary table 2**

The list of identified differentially expressed proteins in different stages of EB6, EB12 and EB20 compared to ESC.

| Down-regulated proteins (>20%, p-value <0.05) | Up-regulated proteins (>20%, p-value <0.05) | Stage |
| --- | --- | --- |
| HIST1H4I,VIM,LGALS1,RPL4,PEA15,SH3BGRL3,RPS24,RPL7,CALM2,LAMA1 ,SSBP1 ,HSPE1 ,ATP5A1 ,ERp31, PGRMC1 , ENSG00000187978 1 ,SLC25A5, ,IPI00455557, ATP5B ,HIST1H2AJ ,PPT1 ,CTSC,RCN2 ,HSPA9 STOML2 ,LMNB1 ,HSP90B1 ,CANX , MCP ,PRDX3 ,HNRNPA2B1 | EEF1B2,GST3,PFKP,IPI00790768,LIN28,SLC2A1 ,PGK1 ,TNC ,VIL2 ,LCP1 ,MDH1, KU80, PDLIM1 ,GPI ,CTSB,TPI1 ,COL18A1 , EEF1A1, KRT8 ,GGH,TSP1 ,CNBP ,NID2, COPE, SLC2A3,TLN1 , SERPINB9 ,IPI00790691, KRT19,HAPLN1 ,EDS4A,LCD1,MTF,MIF ,ALB | EB6 |
| HIST1H4I,LGALS1 ,RPL4 ,RPS24 ,VIM ,RPL7,PPT1 ,L1TD1 ,SSBP1 ,HSPE1 ,ENSG00000187978 1 ,PEA15,LAMA1 ,IPI00455557,NCL,UQCRC1,SH3BGRL3 ,DDX5 , ATP5B ,G3BP1 ,CTSC,HIST1H2AJ ,GRMC1 ,VDAC1 ,SLC25A5 ,ATP5A1 ,HNRPA1 ,HET,RBMX ,PDIA6 , ATP1A1 ,RPN1 ,RCN ,HSPA9 ,ERp31,HSP90B1 ,CCT2 ,HNRPAB ,HNRPDL ,RPLP2 ,EEF1D ,RPS7 ,RPS19 ,RSE1 | FUS,KU80,HNRPH1,IPI00480131,PGD ,GST3,PGK1 , ENO1 ,RAB2 ,FLN2 ,GSN ,DSP,KRT7 ,PARK7,TPI1 ,ANXA1 ,IPI00555610,HSPB1 ,SNL,MSN ,NUDT21 ,GPI ,CKB, EEF1A1, KRT18 ,TSP1 , ANXA5 ,CORO1C ,VIL2 ,CTSB, KRT8 ,COL18A1,SLC2A1 EDS4A, GGH, NID2, LCD1, SERPINB9 ,IPI00790691,COPE,SLC2A3,KRT19,LCP1 ,ALDH2 , HAPLN1 , MTF, ALB ,MIF | EB12 |
| HIST1H4I,CALM2,L1TD1 ,PEA15,LAMB1 ,LAMA1 , NID1,NASP,CTSC,RPS24 ,ATP5A1 ,HNRNPA2B1 ,HSPE1 , XRN2 ,LIN28,PSPC1,G3BP1 ,LAMC1 ,HIST1H2AJ , ENSG00000187978 1 ,IPI00455557,SNRPD2,LGALS1 , RPL4 ,RPL7,SSBP1 ,ADAR ,BANF1, IPI00783983 ,ATQ1, PSIP1 ,PGRMC1 ,HSPA9 ,NCL,HNRPDL ,RANBP5 ,IGF2BP3 , HNRPC,RPS19 ,DDX5 ,HC8,YWHAQ ,SRG3 ,HET, CTNNB , PRP,ATP5B ,RCN2 ,PTB,UQCRC1,RBMX ,VDAC1 ,SFPQ HNRPAB ,RPS7 ,IDH1 ,SFRS3,RPN1 ,IPI00796199, HNRPA1 ,RPL23 | ANXA5,ACTG1,GST3,MAP1B ,ACTN4 ,PPIB ,NPC2 ,FLNA ,KPNB1 ,CORO1C ,MTF,MYL6 ,P63,CALU ,PARK7,AK2 ,MYH9 , DSP,COL18A1,CD49B,P4HB ,SERPINB9 ,ACTN1 , RDX, ITGB1 ,HAPLN1 ,RCN ,EEF1B2 ,TPM4 , SNL,EEF1A1, IPI00647915,SLC3A2,RAB2 ,TNC ,VIL2 ,PDLIM , IPI00480131, MYL9 ,FLN2 ,MIF ,ANXA1 ,PFKP ,GGH, PDLIM1 , CTSB,IPI00555610,LCD1,NID2,CALD1 ,IP2 ,HSPB1 , TGM2 ,ALDH2,COPE,KRT7 ,ALB ,KRT8 ,SLC2A3, IPI00790691,KRT18 ,LC2A1 KRT19,LCP1, EDS4A,S100A10 ,TSP1 | EB20 |
