# Supplementary material for: Quantitative Proteomic Analysis of Human Embryonic Stem Cell Differentiation by 8-Plex iTRAQ Labelling
Source: PLoS One. 2012 Jun 18;7(6):e38532. doi: 10.1371/journal.pone.0038532 (PMC3377673; doi:10.1371/journal.pone.0038532)
Supplement: Table S4 — List of the proteins depicted in the figure 2, with their expression ratio and p-values. (DOCX) [file pone.0038532.s006.docx]

**Supplementary table 4:** List of the proteins depicted in the figure 2, with their expression ratio and p-values.

| Fold change | < 2^-1^ | 2^-1^ - 1.2^-1^ | 1.2^-1^ - 1.2 | 1.2 - 2 | 2< |
| --- | --- | --- | --- | --- | --- |
| Ratio interval | (-∞,0.500] | [0.500,0.833] | [0.833,1.200] | [1.200,2.000] | [2.000,∞) |
| Representative color |  |  |  |  |  |

| **G1** | **Gene symbol** | **EB6 /ESC** | **P-value** | **EB12/ESC** | **P-value** | **EB20/ESC** | **P-value** |
| --- | --- | --- | --- | --- | --- | --- | --- |
| 1 | RPL23 | 0.883 | 0.037 | 0.879 | 0.048 | 0.831 | 0.003 |
| 2 | PEA15 | 0.612 | 0.009 | 0.689 | 0.056 | 0.528 | 0.003 |
| 3 | HSPA9 | 0.808 | 0.023 | 0.795 | 0.049 | 0.748 | 5E-05 |
| 4 | ATP5B | 0.78 | 9E-04 | 0.734 | 1E-04 | 0.773 | 2E-04 |
| 5 | HIST1H4I | 0.522 | 5E-04 | 0.499 | 2E-04 | 0.46 | 5E-04 |
| 6 | ENSG00000187978 1 | 0.75 | 3E-04 | 0.685 | 1E-04 | 0.696 | 0.001 |
| 7 | HNRPA1 | 0.925 | 0.008 | 0.772 | 0.041 | 0.828 | 0.001 |
| 8 | NCL | 0.864 | 0.039 | 0.709 | 0.001 | 0.748 | 0.002 |
| 9 | HSPE1 | 0.727 | 0.018 | 0.664 | 0.003 | 0.652 | 0.002 |
| 10 | RPL4 | 0.589 | 0.001 | 0.577 | 0.002 | 0.704 | 0.003 |
| 11 | RPS24 | 0.67 | 0.022 | 0.599 | 0.002 | 0.63 | 0.003 |
| 12 | LGALS1 | 0.573 | 0.003 | 0.577 | 8E-04 | 0.702 | 0.004 |
| 13 | DDX5 | 0.856 | 0.142 | 0.734 | 0.023 | 0.755 | 0.006 |
| 14 | SSBP1 | 0.727 | 0.002 | 0.639 | 3E-04 | 0.712 | 0.006 |
| 15 | RPL7 | 0.678 | 0.03 | 0.612 | 4E-04 | 0.707 | 0.009 |
| 16 | HNRPAB | 0.835 | 0.02 | 0.804 | 0.038 | 0.804 | 0.011 |
| 17 | VDAC1 | 0.849 | 0.127 | 0.765 | 0.006 | 0.799 | 0.012 |
| 18 | PGRMC1 | 0.749 | 0.002 | 0.756 | 0.002 | 0.745 | 0.012 |
| 19 | HET | 0.838 | 0.239 | 0.778 | 0.034 | 0.767 | 0.013 |
| 20 | RPN1 | 0.906 | 0.539 | 0.793 | 0.011 | 0.819 | 0.016 |
| 21 | CANX | 0.82 | 0.001 | 0.855 | 0.047 | 0.853 | 0.019 |
| 22 | CCT2 | 0.892 | 0.072 | 0.803 | 0.041 | 0.835 | 0.022 |
| 23 | HIST1H2AJ | 0.785 | 0.284 | 0.748 | 0.168 | 0.695 | 0.03 |
| 24 | RPS7 | 0.909 | 0.341 | 0.825 | 0.108 | 0.809 | 0.035 |
| 25 | IPI00455557 | 0.764 | 0.022 | 0.706 | 0.013 | 0.697 | 0.05 |
| 26 | UQCRC1 | 0.876 | 0.216 | 0.721 | 0.01 | 0.795 | 0.241 |
| 27 | STOML2 | 0.809 | 0.005 | 0.942 | 0.667 | 0.882 | 0.149 |
| 28 | HNRPC | 0.87 | 0.036 | 0.864 | 0.016 | 0.752 | 5E-04 |
| 29 | CTSC | 0.794 | 0.025 | 0.74 | 0.025 | 0.602 | 6E-04 |
| 30 | L1TD1 | 0.854 | 0.112 | 0.625 | 2E-05 | 0.511 | 2E-05 |
| 31 | G3BP1 | 0.953 | 0.647 | 0.735 | 0.006 | 0.679 | 0.005 |
| 32 | IDH1 | 0.929 | 0.205 | 0.863 | 0.006 | 0.815 | 0.027 |
| 33 | RBMX | 1 | 0.995 | 0.778 | 0.104 | 0.799 | 0.047 |
| 34 | RCN2 | 0.795 | 0.004 | 0.917 | 0.251 | 0.779 | 0.092 |
| 35 | NPM1 | 0.826 | 0.033 | 0.825 | 0.065 | 0.819 | 0.073 |
| 36 | ATP5A1 | 0.73 | 0.002 | 0.77 | 2E-05 | 0.63 | 2E-05 |
| 37 | LAMA1 | 0.698 | 0.093 | 0.701 | 0.091 | 0.536 | 0.002 |

Supplementary Table 4

| **G2** | **Gene symbol** | **EB6 /ESC** | **P-value** | **EB12/ESC** | **P-value** | **EB20/ESC** | **P-value** |
| --- | --- | --- | --- | --- | --- | --- | --- |
| 1 | NASP | 1.09 | 0.554 | 1.052 | 0.775 | 0.592 | 0.01 |
| 2 | SNRPD2 | 1.088 | 0.486 | 1.014 | 0.894 | 0.7 | 0.016 |
| 3 | LIN28 | 1.21 | 0.113 | 1.104 | 0.498 | 0.669 | 0.024 |
| 4 | SRG3 | 1.007 | 0.867 | 1.015 | 0.844 | 0.762 | 9E-04 |
| 5 | ATQ1 | 0.993 | 0.952 | 1.042 | 0.509 | 0.739 | 0.009 |
| 6 | ADAR | 0.992 | 0.935 | 1.077 | 0.573 | 0.726 | 0.04 |
| 7 | YWHAQ | 1.045 | 0.668 | 1.087 | 0.485 | 0.759 | 0.045 |
| 8 | NID1 | 0.987 | 0.957 | 0.864 | 0.473 | 0.59 | 0.007 |
| 9 | IPI00783983 | 1.044 | 0.669 | 0.884 | 0.357 | 0.737 | 0.026 |
| 10 | PSPC1 | 1.09 | 0.681 | 0.836 | 0.057 | 0.673 | 0.039 |
| 11 | PRP | 1.029 | 0.849 | 0.933 | 0.473 | 0.77 | 0.047 |
| 12 | HNRNPA2B1 | 0.833 | 8E-04 | 0.844 | 0.028 | 0.641 | 5E-06 |
| 13 | LAMB1 | 0.935 | 0.784 | 0.866 | 0.469 | 0.534 | 0.003 |
| 14 | CTNNB | 0.963 | 0.776 | 0.947 | 0.404 | 0.769 | 0.036 |
| 15 | RANBP5 | 0.952 | 0.569 | 0.863 | 0.13 | 0.751 | 0.002 |
| 16 | HNRPDL | 1.032 | 0.227 | 0.816 | 0.06 | 0.75 | 0.007 |
| 17 | IGF2BP3 | 0.916 | 0.529 | 0.858 | 0.459 | 0.752 | 0.009 |
| 18 | PSIP1 | 0.914 | 0.2 | 0.862 | 0.178 | 0.74 | 0.011 |
| 19 | RPS19 | 1.016 | 0.906 | 0.827 | 0.003 | 0.755 | 0.065 |
| 20 | SFRS3 | 0.92 | 0.112 | 0.957 | 0.413 | 0.817 | 8E-04 |
| 21 | PTB | 0.898 | 0.255 | 0.936 | 0.469 | 0.783 | 0.005 |
| 22 | BANF1 | 0.845 | 0.064 | 0.878 | 0.047 | 0.735 | 0.019 |
| 23 | CALM2 | 0.682 | 0.001 | 0.979 | 0.944 | 0.467 | 0.008 |
| 24 | IPI00796199 | 0.947 | 0.501 | 1.018 | 0.456 | 0.822 | 0.01 |
| 25 | HC8 | 0.952 | 0.396 | 1.036 | 0.831 | 0.757 | 0.01 |
| 26 | XRN2 | 0.916 | 0.464 | 0.938 | 0.508 | 0.659 | 0.023 |
| 27 | LAMC1 | 0.937 | 0.739 | 0.972 | 0.861 | 0.681 | 0.032 |
| 28 | SFPQ | 0.904 | 0.231 | 1.024 | 0.838 | 0.802 | 0.033 |
| 29 | HSC70 | 0.972 | 0.586 | 0.903 | 0.030 | 0.829 | 0.007 |
| 30 | MCP | 0.824 | 0.045 | 1.019 | 0.595 | 0.865 | 0.049 |

Supplementary Table 4. Continued.

| **G3** | **Gene symbol** | **EB6 /ESC** | **P-value** | **EB12/ESC** | **P-value** | **EB20/ESC** | **P-value** |
| --- | --- | --- | --- | --- | --- | --- | --- |
| 1 | RSE1 | 1.008 | 0.938 | 0.83 | 0.026 | 1.079 | 0.431 |
| 2 | RPLP2 | 0.953 | 0.476 | 0.817 | 0.004 | 1.026 | 0.274 |
| 3 | ATP1A1 | 0.877 | 0.177 | 0.79 | 0.009 | 1.047 | 0.363 |
| 4 | PPT1 | 0.789 | 0.037 | 0.618 | 0.009 | 0.87 | 0.599 |
| 5 | EEF1D | 0.964 | 0.368 | 0.821 | 0.01 | 1.015 | 0.791 |
| 6 | PDIA6 | 0.85 | 0.012 | 0.79 | 0.012 | 0.937 | 0.36 |
| 7 | VIM | 0.54 | 3E-04 | 0.6 | 2E-05 | 1.047 | 0.182 |
| 8 | HSP90B1 | 0.816 | 5E-04 | 0.803 | 4E-05 | 0.958 | 0.384 |
| 9 | SH3BGRL3 | 0.646 | 0.014 | 0.732 | 0.004 | 1.011 | 0.946 |
| 10 | SLC25A5 | 0.753 | 0.073 | 0.769 | 0.022 | 0.912 | 0.436 |
| 11 | PRDX3 | 0.828 | 0.005 | 0.848 | 0.03 | 1.018 | 0.807 |
| 12 | ERp31 | 0.745 | 4E-04 | 0.801 | 0.046 | 0.923 | 0.616 |
| 13 | LMNB1 | 0.813 | 0.003 | 0.903 | 0.095 | 0.966 | 0.723 |

| **G4** | **Gene symbol** | **EB6 /ESC** | **P-value** | **EB12/ESC** | **P-value** | **EB20/ESC** | **P-value** |
| --- | --- | --- | --- | --- | --- | --- | --- |
| 1 | COL18A1 | 1.307 | 0.127 | 1.503 | 0.06 | 1.386 | 0.016 |
| 2 | LCD1 | 1.617 | 0.177 | 1.699 | 0.14 | 1.692 | 0.038 |
| 3 | GST3 | 1.207 | 0.029 | 1.223 | 0.036 | 1.223 | 0.051 |
| 4 | CORO1C | 1.173 | 0.134 | 1.466 | 0.262 | 1.266 | 0.025 |
| 5 | NUDT21 | 1.094 | 0.531 | 1.329 | 0.006 | 1.058 | 0.833 |
| 6 | PGD | 1.102 | 0.381 | 1.212 | 0.008 | 1.115 | 0.161 |
| 7 | CKB | 1.112 | 0.275 | 1.333 | 0.015 | 1.12 | 0.413 |
| 8 | GSN | 1.017 | 0.392 | 1.26 | 0.044 | 1.054 | 0.502 |
| 9 | ALB | 2.766 | 0.058 | 2.326 | 0.112 | 2.155 | 0.002 |
| 10 | PGK1 | 1.234 | 0.001 | 1.232 | 6E-04 | 1.123 | 0.004 |
| 11 | MDH1 | 1.255 | 0.042 | 1.189 | 0.006 | 1.105 | 0.284 |
| 12 | KU80 | 1.256 | 0.002 | 1.202 | 0.015 | 1.104 | 0.11 |
| 13 | SERPINB9 | 1.434 | 0.027 | 1.703 | 0.015 | 1.393 | 0.074 |
| 14 | TPI1 | 1.302 | 0.004 | 1.301 | 0.016 | 1.125 | 0.07 |
| 15 | MTF | 1.79 | 0.238 | 2.209 | 0.02 | 1.282 | 0.187 |
| 16 | HAPLN1 | 1.605 | 0.061 | 2.001 | 0.022 | 1.413 | 0.077 |
| 17 | MIF | 1.825 | 0.172 | 2.393 | 0.036 | 1.605 | 0.177 |
| 18 | MSN | 1.182 | 0.083 | 1.328 | 0.038 | 1.074 | 0.227 |
| 19 | IPI00790768 | 1.209 | 0.025 | 1.172 | 0.174 | 1.167 | 0.13 |
| 20 | GPI | 1.273 | 0.03 | 1.329 | 0.088 | 1.169 | 0.093 |
| 21 | STMN1 | 1.453 | 0.152 | 1.294 | 0.041 | 1.047 | 0.389 |
| 22 | ANXA5 | 0.992 | 0.849 | 1.409 | 0.224 | 1.206 | 3E-04 |

Supplementary Table 4. Continued.

| **G5** | **Gene symbol** | **EB6 /ESC** | **P-value** | **EB12/ESC** | **P-value** | **EB20/ESC** | **P-value** |
| --- | --- | --- | --- | --- | --- | --- | --- |
| 1 | S100A10 | 1.147 | 0.579 | 1.185 | 0.152 | 2.69 | 0.008 |
| 2 | CD49B | 1.085 | 0.281 | 1.014 | 0.897 | 1.391 | 0.008 |
| 3 | NPC2 | 1.138 | 0.208 | 0.95 | 0.755 | 1.249 | 0.024 |
| 4 | MYH9 | 1.001 | 0.986 | 0.988 | 0.728 | 1.345 | 4E-05 |
| 5 | P4HB | 0.934 | 0.291 | 0.958 | 0.526 | 1.393 | 2E-04 |
| 6 | FLNA | 0.949 | 0.014 | 0.949 | 0.086 | 1.252 | 8E-04 |
| 7 | MYL6 | 0.906 | 0.001 | 0.93 | 0.053 | 1.301 | 9E-04 |
| 8 | IPI00647915 | 0.926 | 0.15 | 0.886 | 0.009 | 1.478 | 0.001 |
| 9 | PPIB | 0.979 | 0.702 | 0.911 | 0.305 | 1.246 | 0.001 |
| 10 | MYL9 | 0.873 | 0.405 | 0.918 | 0.558 | 1.576 | 0.001 |
| 11 | ACTG1 | 0.91 | 0.035 | 0.906 | 0.022 | 1.223 | 0.002 |
| 12 | ACTN1 | 0.85 | 0.027 | 0.939 | 0.487 | 1.405 | 0.003 |
| 13 | PDLIM7 | 0.837 | 0.053 | 0.918 | 0.268 | 1.514 | 0.003 |
| 14 | ITGB1 | 0.926 | 0.175 | 1.033 | 0.683 | 1.412 | 0.004 |
| 15 | TGM2 | 0.898 | 0.258 | 0.977 | 0.633 | 1.769 | 0.006 |
| 16 | P63 | 1.013 | 0.758 | 1.005 | 0.969 | 1.306 | 0.009 |
| 17 | CALU | 0.939 | 0.488 | 0.992 | 0.909 | 1.331 | 0.011 |
| 18 | ACTN4 | 0.95 | 0.339 | 1.048 | 0.572 | 1.238 | 0.015 |
| 19 | RCN | 0.841 | 0.175 | 0.794 | 0.011 | 1.418 | 0.033 |
| 20 | CALD1 | 0.955 | 0.573 | 1.077 | 0.633 | 1.719 | 0.034 |
| 21 | TPM4 | 0.928 | 0.347 | 1.014 | 0.922 | 1.428 | 0.043 |
| 22 | KRT18 | 1.096 | 0.234 | 1.349 | 0.048 | 2.284 | 2E-04 |
| 23 | KRT7 | 1.134 | 0.06 | 1.288 | 0.023 | 2.038 | 3E-04 |
| 24 | LIP2 | 1.106 | 0.197 | 1.142 | 0.162 | 1.721 | 0.018 |
| 25 | TSP1 | 1.349 | 0.015 | 1.395 | 0.116 | 2.819 | 0.034 |
| 26 | EEF1B2 | 1.201 | 0.172 | 1.065 | 0.194 | 1.426 | 0.003 |
| 27 | PDLIM1 | 1.264 | 0.271 | 1.032 | 0.853 | 1.645 | 0.01 |
| 28 | KPNB1 | 1.196 | 0.164 | 0.989 | 0.941 | 1.262 | 0.048 |

Supplementary Table 4. Continued.

| **G6** | **Gene symbol** | **EB6 /ESC** | **P-value** | **EB12/ESC** | **P-value** | **EB20/ESC** | **P-value** |
| --- | --- | --- | --- | --- | --- | --- | --- |
| 1 | KRT19 | 1.526 | 0.008 | 1.795 | 1E-04 | 2.371 | 6E-04 |
| 2 | SNL | 1.11 | 0.121 | 1.32 | 0.003 | 1.45 | 6E-04 |
| 3 | EDS4A | 1.607 | 0.081 | 1.557 | 0.065 | 2.403 | 0.002 |
| 4 | SLC2A3 | 1.418 | 0.044 | 1.789 | 0.019 | 2.228 | 0.003 |
| 5 | COPE | 1.376 | 0.536 | 1.765 | 0.129 | 2.022 | 0.009 |
| 6 | DSP | 1.153 | 0.014 | 1.278 | 0.006 | 1.361 | 0.033 |
| 7 | IPI00790691 | 1.45 | 0.138 | 1.762 | 0.049 | 2.247 | 0.034 |
| 8 | CTSB | 1.287 | 0.019 | 1.477 | 0.006 | 1.646 | 0.044 |
| 9 | IPI00480131 | 1.051 | 0.073 | 1.21 | 0.008 | 1.523 | 8E-06 |
| 10 | KRT8 | 1.316 | 0.009 | 1.477 | 4E-04 | 2.221 | 2E-04 |
| 11 | LCP1 | 1.253 | 0.111 | 1.842 | 0.022 | 2.38 | 7E-04 |
| 12 | RDX | 1.087 | 0.37 | 1.167 | 0.395 | 1.408 | 8E-04 |
| 13 | HSPB1 | 1.068 | 0.461 | 1.319 | 0.015 | 1.764 | 0.001 |
| 14 | IPI00555610 | 1.074 | 0.16 | 1.315 | 0.001 | 1.646 | 0.001 |
| 15 | ANXA1 | 1.052 | 0.634 | 1.307 | 0.008 | 1.609 | 0.002 |
| 16 | PFKP | 1.208 | 0.012 | 1.192 | 0.027 | 1.625 | 0.009 |
| 17 | RAB2 | 1.102 | 0.129 | 1.246 | 0.026 | 1.481 | 0.01 |
| 18 | SLC3A2 | 1.008 | 0.924 | 1.172 | 0.222 | 1.479 | 0.016 |
| 19 | AK2 | 1.058 | 0.589 | 1.192 | 0.27 | 1.339 | 0.017 |
| 20 | MAP1B | 1.031 | 0.561 | 1.083 | 0.297 | 1.232 | 0.033 |
| 21 | FLN2 | 1.159 | 0.292 | 1.247 | 0.066 | 1.595 | 0.033 |
| 22 | SLC2A1 | 1.221 | 0.05 | 1.533 | 0.015 | 2.325 | 0.036 |
| 23 | TNC | 1.242 | 0.153 | 1.15 | 0.111 | 1.492 | 0.005 |
| 24 | EEF1A1 | 1.315 | 0.132 | 1.349 | 0.036 | 1.466 | 0.003 |
| 25 | PARK7 | 1.126 | 0.38 | 1.292 | 0.068 | 1.338 | 0.009 |
| 26 | VIL2 | 1.249 | 0.089 | 1.467 | 0.003 | 1.514 | 0.016 |
| 27 | GGH | 1.316 | 0.078 | 1.609 | 0.009 | 1.636 | 0.026 |
| 28 | ALDH2 | 1.178 | 0.313 | 1.89 | 0.012 | 1.848 | 0.05 |
| 29 | NID2 | 1.372 | 0.064 | 1.619 | 0.008 | 1.694 | 0.057 |

Supplementary Table 4. Continued.
